# Basal Forebrain Nuclei Display Distinct Projecting Pathways and Functional Circuits to Sensory Primary and Prefrontal Cortices in the Rat

**DOI:** 10.3389/fnana.2018.00069

**Published:** 2018-08-15

**Authors:** Irene Chaves-Coira, Jesús Martín-Cortecero, Angel Nuñez, Margarita L. Rodrigo-Angulo

**Affiliations:** Departamento de Anatomía, Histología y Neurociencia, Facultad de Medicina, Universidad Autónoma de Madrid, Madrid, Spain

**Keywords:** diagonal band nuclei of Broca, basal magnocellular nucleus, cholinergic neurons, medial prefrontal cortex, optogenetic stimulation

## Abstract

Recent evidence supports that specific projections between different basal forebrain (BF) nuclei and their cortical targets are necessary to modulate cognitive functions in the cortex. We tested the hypothesis of the existence of specific neuronal populations in the BF linking with specific sensory, motor, and prefrontal cortices in rats. Neuronal tracing techniques were performed using retrograde tracers injected in the primary somatosensory (S1), auditory (A1), and visual (V1) cortical areas, in the medial prefrontal cortex (mPFC) as well as in BF nuclei. Results indicate that the vertical and horizontal diagonal band of Broca (VDB/HDB) nuclei target specific sensory cortical areas and maintains reciprocal projections with the prelimbic/infralimbic (PL/IL) area of the mPFC. The basal magnocellular nucleus (B nucleus) has more widespread targets in the sensory-motor cortex and does not project to the PL/IL cortex. Optogenetic stimulation was used to establish if BF neurons modulate whisker responses recorded in S1 and PL/IL cortices. We drove the expression of high levels of channelrhodopsin-2, tagged with a fluorescent protein (ChR2-eYFP) by injection of a virus in HDB or B nuclei. Blue-light pulses were delivered to the BF through a thin optic fiber to stimulate these neurons. Blue-light stimulation directed toward the HDB facilitated whisker responses in S1 cortex through activation of muscarinic receptors. The same optogenetic stimulation of HDB induced an inhibition of whisker responses in mPFC by activation of nicotinic receptors. Blue-light stimulation directed toward the B nucleus had lower effects than HDB stimulation. Our findings pointed the presence of specific neuronal networks between the BF and the cortex that may play different roles in the control of cortical activity.

## Introduction

The mammalian cerebral cortex receives consistent projections from cholinergic and non-cholinergic neurons located in the basal forebrain (BF). The BF includes different structures and nuclei: the medial septum, the horizontal and vertical limbs of the diagonal band of Broca (HDB and VDB, respectively), the substantia innominata, and the nucleus basalis magnocellularis (B nucleus, or in humans, Meynert basal magnocellular nucleus). These structures provide most of the cholinergic innervation to sensory, motor, and prefrontal cortices and hippocampus (Houser et al., 1985; Semba and Fibiger, 1989; Mesulam et al., 1992; Gritti et al., 1997; Semba, 2000; Bloem et al., 2014; Zaborszky et al., 2015; Kim et al., 2016; for review see Butcher and Woolf, 2004). There is abundant evidence that BF cortical projections play an essential role in cognitive functions such as attention states, learning and memory, wakefulness and processing of sensory information (e.g., Manns et al., 2001; Broussard et al., 2009; Sarter et al., 2014; Zant et al., 2016; for review see Hasselmo and Sarter, 2011).

Given its prominent role in regulating attention and memory neural circuits, the cholinergic signaling is a key player in mediating cognitive performance. The cholinergic hypothesis of Alzheimer’s disease (AD) emerged from evidences of loss of cholinergic markers in the cortex (Geula and Mesulam, 1989), loss of the number of neurons in the BF (Whitehouse et al., 1982), and the report of a volume loss in the nucleus basalis of Meynert in mild cognitive impairment patients (prodromal signs of AD; Cantero et al., 2016). Although AD is most classically associated with memory deficits, these deficits are typically conflated with attentional issues (Romberg et al., 2013). Nevertheless, both sensory-motor deficits and arousal decline appear later in AD patients and may be due to BF-cortical pathways.

Anatomical studies have indicated the existence of a highly structured and topographic organization of BF efferent projections to sensory cortices that may support the well-known cognitive functions of BF neurons (Zaborszky, 2002; Zaborszky et al., 2005, 2015). The above-mentioned authors propose that cholinergic and non-cholinergic projections to the neocortex are not diffuse but are instead organized into segregated or overlapped neuronal groups. In addition, the rostro caudal distribution of retrogradely labeled neurons in BF shows that neurons projecting to the medial prefrontal cortex (mPFC) tend to cluster in the rostral portion of the BF (Chandler et al., 2013). Cholinergic pathways originated in different BF nuclei have been described following four different routes, depending on their location, to reach different layers of the mPFC (Bloem et al., 2014). Consistent with these findings, optogenetic activation of cholinergic neurons in BF induces modality-selective desynchronization in specific sensory cortices (Kim et al., 2016) and sensory modulation in mice (Chaves-Coira et al., 2016).

Despite many studies on the relations between the location of different BF nuclei and their projection cortical areas, their role in sensory processing remains unanswered. The aim of the present work is to determine the existence of specific neuronal populations in the BF linking with specific sensory, motor and prefrontal cortices in rats. Neuronal tracing techniques combined with sensory and optogenetic stimulations have been used to determine the existence of specific neuronal networks that may play different functions in cortical information processing.

## Materials and Methods

### Animals

Anatomical experiments were performed in 59 adult Sprague-Dawley rats weighing 220–290 g (**Table 1**). Animals were housed under standard colony conditions and food and water were supplied *ad libitum*. In accordance with European Community Council Directive 2010/63/UE all animal procedures were approved by the Ethical Committee of the *Universidad Autónoma de Madrid* (CEI72-1286-A156). Efforts were made to minimize animal suffering as well as to reduce the number of animals used.

**Table 1:** Number of animals injected in the different structures with fluorescent tracers and viral vector.

	S1 + A1	S1 + V1	Il/Pl	M2	HDB	B	HDB + B
FlGo			9	8			
FB							
FlGo + FB	10	11					11
Viral vector + optogenetic					5	5	


### Anatomical Procedures

The neuroanatomical fluorescent retrograde tracers Fluoro-Gold (FG; Fluorochrome, LLC, Denver, CO, United States) and Fast Blue (FB; Polysciences, Inc., Warrington, PA, United States) were used for the experiments. Animals were anesthetized with an intraperitoneal injection of ketamine (70 mg/kg) plus xylazine (5 mg/kg) before being placed in a stereotaxic frame for appropriate craniotomy; supplementary doses of the anesthetic were applied when it was necessary (35 mg/kg and 2.5 mg/kg, respectively; i.p.). The analgesic Metacam (meloxicam 1 mg/kg; s.c.) was also administered to the animals at the end of the surgery.

### Injections of Fluorescent Retrograde Tracers in Cortical Areas

Employing coordinates from the Paxinos and Watson (2007) different methodological approaches were taken. In order to elucidate the anatomical pathways linking the BF nuclei with cortical areas, 21 rats received 4% FG injections in S1 and FB deposits (2 mm^2^ of absorbable gelatin “Spongostan” embedded in 1% saline solution) in either the A1 or primary visual (V1) cortices, respectively. In all 21 animals, 20 nl of FG solution was injected through a 0.5 μl Hamilton syringe (10 nl per minute) in S1 at the following stereotaxic coordinates: antero-posterior, -2.3 mm from Bregma; lateral, 5.5 mm and vertical, 1 mm. FB deposits were placed for 20 min in 10 of the 21 animals in A1 at the following stereotaxic coordinates: antero-posterior, -5.8 mm from Bregma; lateral, 7.0 mm and vertical, 1 mm; in the remaining 11 animals, FB deposits were placed in V1 at the following stereotaxic coordinates: antero-posterior, -6.3 mm from Bregma; lateral, 3.5 mm and vertical, 0.5 mm (**Table 1**).

In order to assess the implication of mPFC in the BF projecting pathways, injections of 20 nl of FG were performed through a Hamilton syringe (10 nl per minute) in prelimbic/infralimbic (PL/IL) cortices in nine rats at coordinates: antero-posterior 3.7 mm from Bregma; lateral 0.5 mm and vertical, 2 mm, or in the secondary motor (M2) cortex in eight rats at coordinates: antero-posterior 3.7 mm from Bregma; lateral 1.0 mm and vertical, 0.5 mm (**Table 1**).

### Injections of Fluorescent Retrograde Tracers in BF Nuclei

To ascertain the presence of reciprocal connections between the HDB and B nuclei with different cortices, 11 animals received 30 nl of FG in HDB (coordinates: antero-posterior, 0.24 mm from Bregma; lateral, 1.5 mm and vertical, 8.8 mm) and 30 nl of FB in the B nucleus (coordinates: antero-posterior, -1.32 mm from Bregma; lateral, 2.8 mm and vertical, 7.2 mm).

Once wounds were sutured, animals were housed in individual cages in accordance with the dimensions required for the species and located in a special post-surgery room at the Veterinary Office. Animals were treated with ibuprofen (Dalsy; 20 mg/cc solution; 3 cc/500 cc of drinking water) for the following days and additional doses of meloxicam, a non-steroidal anti-inflammatory drug (Metacam; 1 mg/kg; s.c.) was also administered when necessary.

After a survival period of 1 week animals were anesthetized with an overdose of the same anesthesia and perfused transcardially with 4% paraformaldehyde in 0.1 M phosphate buffer at pH 7.3 followed by increasing concentrations of sucrose solutions (5%, 10%, 20%) in the same buffer. Brains were stored in 30% sucrose for at least 3 days for tissue cryopreservation to be frozen sectioned on the coronal plane at 40 μm. Sections were collected in three consecutively ordered series devoted to Nissl staining, fluorescent visualization and choline acetyltransferase (ChAT) immunostaining series. The series processed for Nissl staining were used for delimiting structures. The series processed for ChAT immunostaining sections were incubated with 1:100 goat anti-ChAT (Chemicon, United States) and with 1:200 Alexa 546 anti-goat (Life Technologies, United States). Sections were mounted on glass slides, dehydrated through passage in ascending grades of alcohol, defatted in xylene for 30–60 min and finally coverslipped with DePeX mounting medium (Serva, Heidelberg, Germany).

Single and double-labeled neurons by the tracers, were studied under both a Nikon Axioskop fluorescent microscope and a confocal microscope. Confocal study was carried out using a Spectral Leica TCS SP5 confocal microscope in which we used a Tile Scan tool of LAS AF software for acquiring the images. FG was excited between 350 and 385 nm and the maximum emission was 530 and 600 nm. FB was excited at 365 nm and the maximum emission was 420 nm. Samples were analyzed using bio-mapping (maximal projections) by sequentially applying both lin405 nm (ultraviolet) laser line for visualization of the fluorescent tracers (FG and FB) and linAr546 nm (applying argon) laser line for visualization of ChAT-positive neurons. In the lin405 nm laser line absorption bars were settled separated enough to distinguish between both fluorescent tracers, FB was acquired using the ultraviolet spectra (410–450 nm) and FG was acquired using the green line (500–560 nm). Non-overlapping checks were done in each case to ensure complete channel separation. Regions of interest were studied using a 10× and 20× objectives, and a 63× oil objective for the quantification of neurons in each channel. Images were a stack of sections in maximal projection which were analyzed in the two channels (ultraviolet and green) and the merged image was also studied. Images shown in the figures are a stack of sections in maximal projection. For the semi quantitative study, neurons were counted in each individual layer of the confocal image. Since we have focused on a qualitative study, for a semi-quantitative analysis the number of single and double-labeled neurons were counted and normalized for each nucleus in each individual animal. The total number of neurons in each nucleus was considered 100% and the percentage of each labeled neurons was calculated. These percentages were used for obtaining the mean percentage of all cases, as is indicated in Results and **Figure 4**.

### Physiological Procedures

Ten rats were anesthetized intraperitoneally with a mixture of ketamine (100 mg/kg) plus xylazine (3 mg/kg) and stereotaxically injected with the adeno-associated viral vector AAV5-CaMKIIα::ChR2(H134R)-eYFP.WPRE.hGH (Addgene26969P, Penn Vector Core, University of North Carolina) into the HDB (*n* = 5) or the B nucleus (*n* = 5) (**Table 1**). We chose this particular vector to drive expression of the light-activated cation channel, channelrhodopsin-2, tagged with a fluorescent protein (ChR2-eYFP) that is specific to Ca^2+^/calmodulin-dependent protein kinase II α (CaMKIIα)-expressing neurons, ensuring that BF neurons anterogradely transport the virus to the cortex (Tye et al., 2011). The coordinates were the same as above mentioned in anatomical procedures. The viral vector was diluted in phosphate buffer to a final concentration of 1.6 × 10^11^ viral particles/ml and 30–100 nl were injected by means of a 0.5 μl Hamilton syringe. Animals were allowed to recover for at least 3 weeks in the animal house before electrophysiology experiments were performed.

To study the effect of optogenetic stimulation of BF neurons on cortical responses, the recovered viral injected animals were anesthetized with urethane (1.6 g/kg; i.p.). The depth of anesthesia was sufficient to eliminate pinch withdrawal, palpebral reflex and whisker movement. Supplemental doses of urethane (0.5 g/kg; i.p.) were administered when required to keep a stable anesthetic level. Animals were placed in a Kopf stereotaxic device (David Kopf Instruments, Tujunga, CA, United States) in which surgical procedures and recordings were performed. The body temperature was maintained at 37°C. Local anesthetic (lidocaine 1%) was applied to all skin incisions. An incision was made exposing the skull and small holes were drilled in the bone at the preselected coordinates.

### Sensory Stimulation

Whisker deflections were performed by brief air puffs using a pneumatic pressure pump (Picospritzer) that delivers an air pulse through a 1 mm inner diameter polyethylene tube (1–2 kg/cm^2^, 20 ms duration, resulting in whisker deflections of ≈15°). To avoid complex responses due to deflections of multiple whiskers, these were trimmed to 5 mm in length so that reproducible responses could be evoked. The experimental protocol consisted of pulses delivered to the principal whisker (whisker that gives the highest spike response) at 1 Hz. We applied 120 whisker stimuli (control period; 2 min). The same pulses were applied after blue light stimulation of the BF during 4 min (240 stimuli).

### Optogenetic Stimulation and Electrophysiological Recordings

Optical stimulation of ChR2-expressing neurons was achieved with light-emitting diode (LED; 473 nm; Thomas Recording, Germany) delivered from an optical fiber (core diameter 120 μm) positioned directly above the HDB or B nucleus. The LED was triggered with a square-step voltage command. Stimulation was applied by a single long-lasting pulse (1 s; 300 ms was the time needed to reach maximum intensity). Illumination intensity was <30 mW/mm^2^ at the BF, which is below the damage threshold of ∼100 mW/mm^2^ for blue light (Cardin et al., 2010).

Field potential recordings were made with tungsten macroelectrodes (<1 MΩ, World Precision Instruments, WPI, Sarasota, FL, United States). The electrical reference consisted in a stainless steel wire that was inserted in the neck musculature. Field potentials were filtered between 0.3 and 100 Hz, amplified and sampled at 500 Hz. Signals were fed into a personal computer with the temporal references of the stimuli for off-line analysis with Spike 2 software (Cambridge Electronic Design, Cambridge, United Kingdom).

At the end of the experiment rats were perfused following the same protocol that was applied in the anatomical studies and brains were sectioned at 40 μm; sections were processed for enhanced version of the yellow moiety of the Aequorea victoria fluorescent protein (eYFP) immunostaining to assess the injection site and electrode position. Following rinses in hydrogen peroxide (2% in PB, 15 min) and Triton X-100 (2% in PB, 30 min), sections were incubated in a purified rabbit antiserum against GFP that also recognizes eYFP (1:500; EXBIO, Prague, Czechia) + 2% Triton X-100 + 3% normal goat serum + 1% bovine serum albumin in PB (16 h, 20°C). After several rinses in PB, sections were incubated in biotinylated goat anti-rabbit IgG (1:100; Sigma-Aldrich, St. Louis, MO, United States) + 2% Triton X-100 + 3% NGS + 1% BSA in PB (2 h, 20°C). Following new rinses in PB, the sections were incubated in avidin-biotin-peroxidase complex solution (1:100; Vectastain Elite, Vector Laboratories, Burlingame, CA, United States) + 2% Triton X-100 (4°C, 16 h). Peroxidase activity was visualized using a glucose oxidase-DAB-nickel protocol (Shu et al., 1988). Sections, were lightly counterstained with Thionin for cytoarchitectonic reference, defatted, dehydrated, and coverslipped with DePeX.

### Drugs

Injections of atropine (5 mg/kg in 0.9% NaCl; i.p.) or mecamylamine hydrochloride (8 mg/kg in 0.9% NaCl; i.p.) were administered to assess if the cholinergic modulation of the cortical responses was due to activation of muscarinic or nicotinic receptors.

### Physiological Data Analysis

The average of the cortical evoked potentials in the S1 cortex and mPFC (triggered by tactile stimuli) were calculated every 1 min (60 stimuli), using Spike 2 software. To perform statistical analysis the area of the evoked potential was measured from the negative slope beginning with the first negative wave up to the same voltage level with a positive slope. The evoked potentials were recorded 2 min before blue light stimulation (control period) and 4 min after the light stimulation.

Statistical analysis was performed using Graph Pad Prism 7 software (San Diego, CA, United States). Results are reported as means ± SEMs. Data were normally distributed, according to the Shapiro–Wilk normality test and analyzed using a Student’s paired *t*-test. Statistical significance was set at a 95% confidence level (two-tailed).

## Results

### Anatomical Pathways Linking BF Nuclei and the Sensory Cortices

Delimitation of BF nuclei was made by studying the Nissl staining series and following the Paxinos and Watson (2007). According to that atlas, HDB extension runs from Bregma +0.8 to Bregma -1.56, we have chosen the coordinates mentioned in “Materials and Methods” since is the region bearing the highest density of neurons. B nucleus extension runs from Bregma -0.36 to Bregma -3.12 of disperse neurons in other structures, it is for that we have chosen the coordinates mentioned in “Materials and Methods.”

**FIGURE 1:**
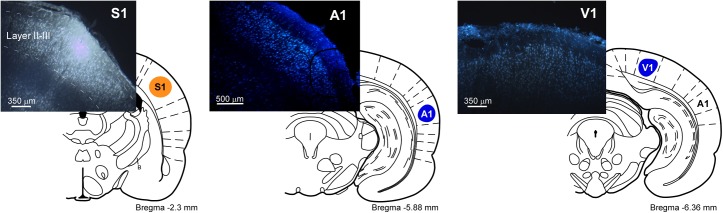
Schematic drawings and microphotographs of brain coronal sections showing the FG injection site in S1 and the FB deposits in A1 and V1.

**FIGURE 2:**
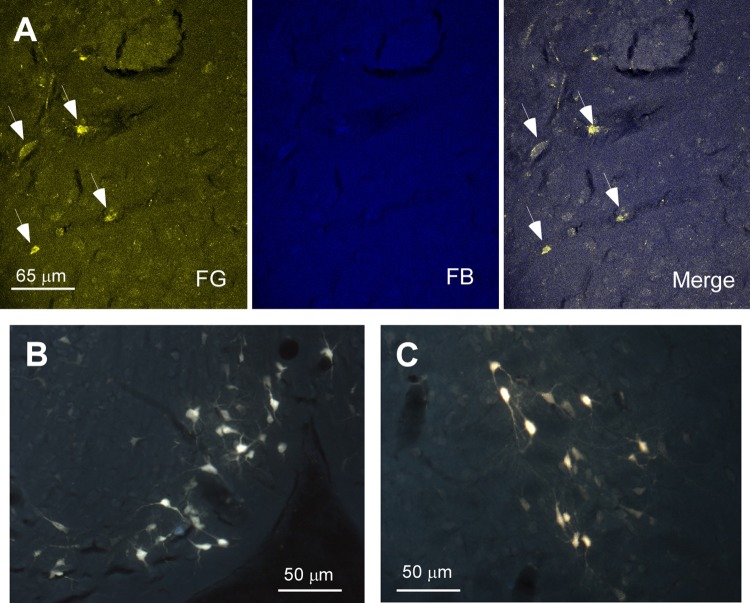
Microphotographs of HDB labeled neurons after injections/deposits in S1/A1 cortices. **(A)** Confocal images of HDB neurons in a representative animal. Note the few neurons stained with FB (deposit in A1). Thus, the merge does not show double-labeled neurons. **(B,C)** Microphotographs under fluorescence microscope showing HDB labeled neurons. Arrows indicate FG labeled neurons.

In order to detect the presence and characteristics of the pathways linking different sensory cortices with specific regions of the BF, simultaneous injections/deposits of FG and FB were performed in S1, A1, and V1 (**Figure 1**). In all cases, the study of the fluorescent and Nissl staining sections confirmed the location of tracers. Ten animals received an FG injection in S1 and a FB deposit in A1. Neurons retrograde labeled with FG, FB or both tracers (double-labeled neurons) were found in different BF regions (**Figure 2**); FG labeled-neurons were located in both HDB/VDB region, in the substantia innominata, at all rostro-caudal levels of the B nucleus, and the zona incerta; however, neurons showing FB fluorescence were found in neither the VDB nor the HDB although they were present at all levels of the B nucleus as well as in the substantia innominata and zona incerta. Most double-labeled-neurons were found in the B nucleus and zona incerta. Quantification of all these neurons allowed us to establish that more than 98% of labeled neurons in the HDB/VDB region were FG single-labeled neurons while the rest (2%) were double-labeled neurons; in the B nucleus 50% of the labeled neurons were single-labeled with FG, 15% single-labeled with FB and 35% double-labeled neurons (see **Figure 4A**). Percentages of this quantitative analysis have been normalized to each injection since the relative number of neurons projecting to S1 cortex was higher than the ones projecting to A1 cortex.

**FIGURE 3:**
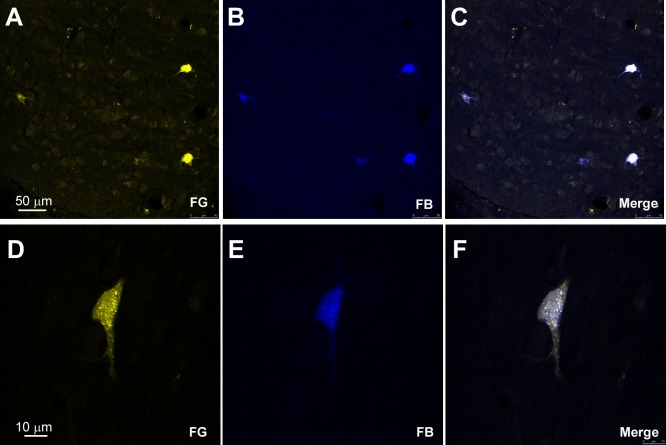
Microphotographs of HDB labeled neurons after injections/deposits in S1/V1. **(A–C)** Confocal images showing single and double-labeled neurons in HDB in a representative animal; the merge shows the presence of double-labeled neurons. **(D–F)** Another examples of double-labeled neurons at high magnification.

**FIGURE 4:**
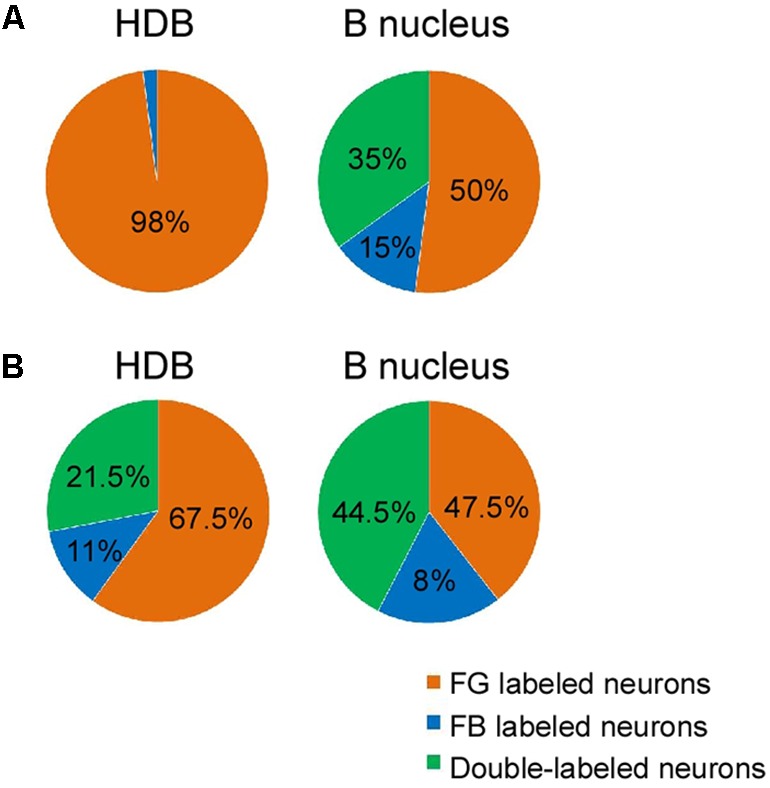
Percentages of single, and double-labeled neurons according to the location of the injection/deposit sites. **(A)** Graphic representation of normalized relative percentages of FG and FB single neurons, and double-labeled neurons found in HDB and B nucleus after injections in S1/A1 cortices. Note that HDB mainly projects to S1 while the B nucleus projects to both S1 and A1 sensory cortices displaying numerous double-labeled neurons. **(B)** Graphic representation of FG and FB single neurons, and double-labeled neurons found in HDB and B nucleus after injections in S1/V1 cortices. B nucleus contained a large proportion of double-labeled neurons, indicating a lower specificity in their cortical projections.

Injections/deposits in the S1 (FG) and in V1 (FB) cortices were performed in 11 animals (**Figure 1**). Single and double-labeled neurons were found in HDB/VDB as well as at all levels of the B nucleus and in the substantia innominata and zona incerta (**Figure 3**). Quantitative studies of labeling in the HDB/VDB showed that 68% of neurons were single-labeled with FG, 11% were single-labeled with FB and 21% of the neurons were double-labeled; in the B nucleus, 47% of the neurons were labeled with FG, 8% with FB and 45% were double-labeled neurons (**Figure 4B**). Percentages of this quantitative analysis have been normalized since the relative number of neurons projecting to S1 cortex was higher than the ones projecting to V1 cortex. All together these data indicated that HDB/VDB showed a majority of single labeled projecting neurons to sensory cortices while B nucleus contained a large proportion of double-labeled neurons, indicating a lower specificity in their cortical projections.

**FIGURE 5:**
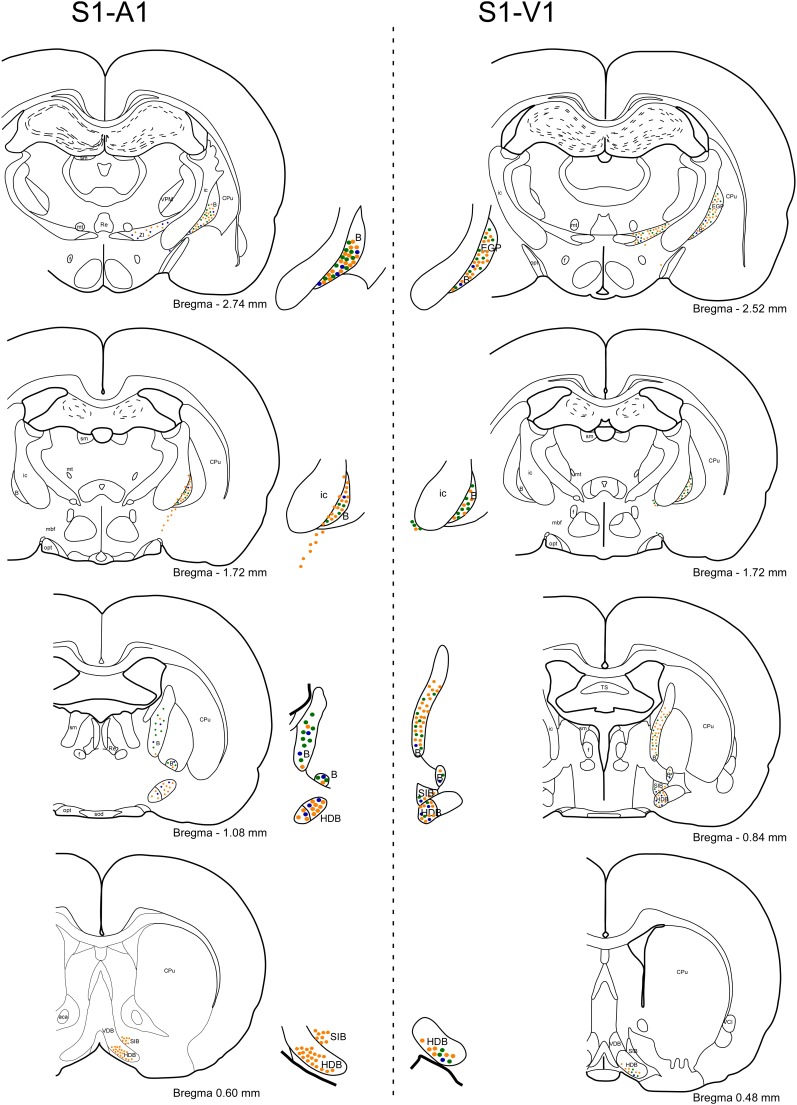
Rostral to caudal schematic drawings of brain coronal sections of BF neurons projecting to S1, A1, and V1 cortices. Neurons projecting to S1 or/and A1 cortices are shown on the left column (o similar) of the figure and to S1 or/and V1 cortices on the right column of the figure. Neurons are depicted as: single-FG (orange, injection in S1), single-FB (blue; injection in A1), single-FB (blue; injection in V1), and double-labeled neurons (green). Notice the different location in the BF nuclei of neurons projecting to S1, A1, or V1 cortices. 3V, 3rd ventricle; aca, anterior commissure; CPu, caudate putamen; EGP, external globus pallidus; Re, reuniens thalamic nucleus; SIB, substantia innominate basal part; SO, supraoptic nucleus; VCl, ventral part of claustrum.

In **Figure 5** labeled neurons from the retrograde tracer injections/deposits in S1/A1 (left column) and S1/V1 (right column) are represented in schematic drawings of brain coronal sections. A rostro-caudal distribution of neurons projecting to S1, A1, and V1 from the different BF nuclei is observed. Neurons projecting from HDB to the three cortices (S1, A1, and V1) were present from the rostral levels all the way to the caudal levels but with a specific pattern. Single labeled and double labeled neurons projecting to S1 and V1 cortices appeared from the very rostral levels of HDB. Single labeled neurons projecting to A1 appeared in a rostro-medial level of HDB, as well as double labeled neurons projecting from HDB to S1/A1. In the case of the labeled neurons projecting from B nucleus to these three cortices, both single and double labeled neurons were found along every rostro-caudal levels studied of the nuclei, in support of a lower specificity of their cortical projections.

**FIGURE 6:**
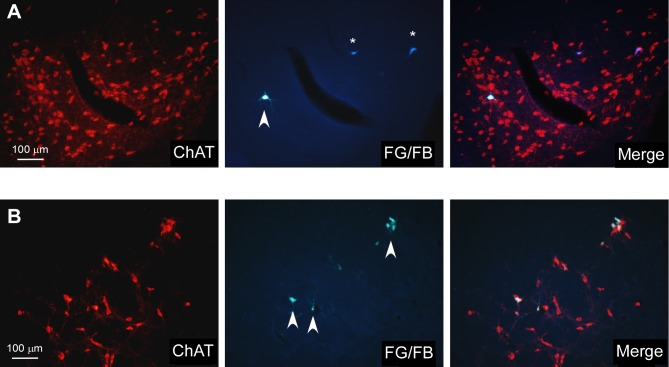
Microphotographs of cholinergic HDB and B labeled neurons after retrograde-trace injections in S1/A1 cortices. **(A)** Images showing ChAT-positive neurons in HDB (left photomicrograph) and FG- (arrowhead; S1 injection) and FB- (asterisks; A1 injection) labeled neurons (central microphotograph); the merge shows that cortical projecting neurons are cholinergic neurons. **(B)** Images showing ChAT-positive neurons in B nucleus (left photomicrograph) and FG-labeled neurons (arrowhead; S1 injection; central photomicrograph); the merge shows that cortical projecting neurons are cholinergic neurons.

Although it is known that BF cholinergic neurons projects to sensory cortices (see for review Butcher and Woolf, 2004), we corroborate this finding by performing a double-labeling of ChAT and FlGo/FB stained neurons (S1/A1 injected cortex, respectively). Representative neurons in HDB or B nuclei are shown in **Figures 6A,B**, respectively. Results indicated that most of projecting neurons to sensory cortices were cholinergic neurons in HDB or B nucleus.

### Anatomical Pathways Linking mPFC and the BF

**FIGURE 7:**
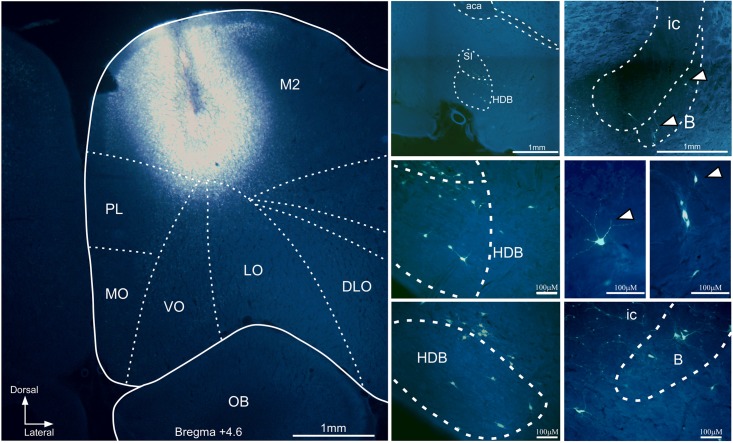
Microphotograph of a coronal section at Bregma +4.6 mm showing the FG injection site in M2 cortex in a representative case **(Left)**. Microphotographs at different magnifications of coronal sections showing the location of FG labeled neurons found in HDB and in B nucleus **(Right)**. DLO, dorsolateral orbital cortex; ic, internal capsule; LO, lateral orbital cortex; MO, medial orbital cortex; OB, olfactory bulb; rest of lettering as in **Figure 5**.

**FIGURE 8:**
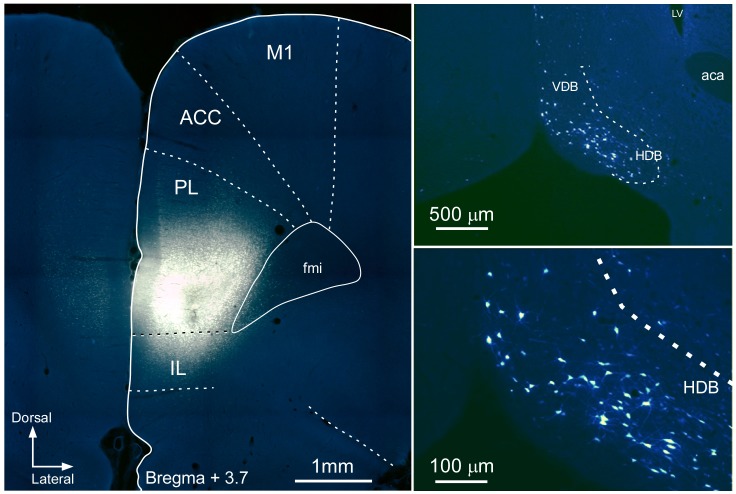
Microphotograph of a coronal section at Bregma +3.72 mm showing the location of the FG injection in PL/IL cortices **(Left)**. Microphotographs at different magnifications of FG labeled neurons in HDB **(Right)**. ACC, anterior cingulate cortex; fmi, corpus callosum forceps; LV, lateral ventricle; M1, primary motor cortex; rest of lettering as in **Figure 5**.

Retrograde tracers were also used to study cortical projections between the mPFC to the BF in 17 rats (**Table 1**). The mPFC is a heterogeneous region. For this reason, we focused our study in the ventral part of the mPFC (prelimbic–infralimbic, PL/IL, cortex) and in the dorsal part of the mPFC (secondary motor, M2) because they are involved in attentional processes and sensory-motor integration (Heidbreder and Groenewegen, 2003; Uylings et al., 2003; Barthas and Kwan, 2017). Injections of FG in the M2 cortex resulted in labeled neurons in HDB/VDB as well as in B nucleus (**Figure 7**) whereas injections of FG in the ventral regions of the mPFC (PL/IL cortices) resulted in a considerable amount of labeled neurons in all rostro-caudal levels of HDB/VDB, while only a few neurons were located in B nucleus (**Figure 8**).

**FIGURE 9:**
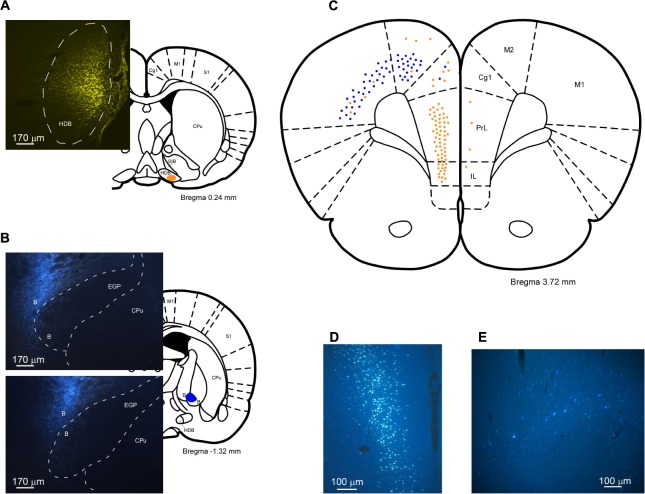
**(A,B)** Schematic drawings and microphotographs of brain coronal sections showing the injection sites in HDB **(A)** and in B nucleus **(B)**. **(C)** Schematic drawing of coronal section at Bregma +3.72 mm showing single-FG (orange), single-FB (blue), and double-labeled (green) neurons. Most of the neurons that project to HDB are located in the PL/IL cortices whereas neurons projecting to B nucleus tend to be located in M2 and anterior cingulate (ACC) cortices. **(D)** An example of labeled neurons in PL/IL cortices is shown. **(E)** An example of neurons labeled in M2 cortex is shown.

To determine if mPFC and BF regions are reciprocally connected FG was injected in HDB nucleus and FB in B nucleus (**Figures 9A,B**, respectively). FG injections in HDB resulted in retrograde labeled-neurons in PL/IL (**Figures 9C,D**). Only a few, scattered neurons were observed in motor cortices. FB injections in B nucleus revealed single, retrograde-labeled neurons in the cingulate and motor cortices (**Figures 9C,E**). Only a few double-labeled neurons appeared and they were confined to motor and cingulate cortices (data not shown). Thus, data indicated the existence of two different pathways between mPFC and the BF; cingulate and motor cortices mainly project to the B nucleus whereas practically all the neurons detected in PL/IL project to the HDB/VDB in a reciprocal projection.

### Sensory Response Modulation by BF Optogenetic Stimulation

**FIGURE 10:**
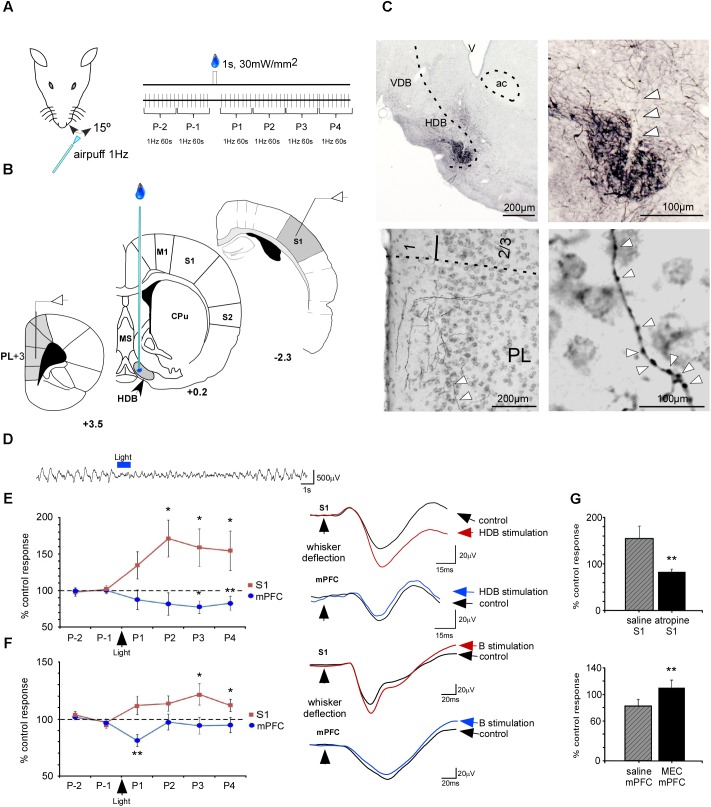
Optogenetic stimulation of BF induced cholinergic modulation of whisker responses in S1 and mPFC cortices. **(A)** Diagram of the experimental protocol. Tactile stimulation (air puffs) was applied to the trimmed contralateral whiskers. **(B)** Schematic drawing of location of stimulating and recording electrodes. **(C)** A representative case of virus injection [AAV5-CaMKIIa-hChR2(H134R)-EYFP] in HDB. Microphotographs of the injection site are shown in the upper part. Arrowheads indicate the cannula track. In the lower part of the figure microphotographs showing fibers and axonal varicosities (arrowheads) are shown. **(D)** A typical effect of HDB light stimulation on the S1 EEG. The light induced an EEG desynchronization. **(E)** Plot of SEP area in controls (2 min) and during the 4 min after HDB blue light stimulation (1 s pulse duration). Note that HDB stimulation induced different simultaneous responses in S1 and mPFC cortices. **(F)** Plot of SEP responses in control (2 min) and during the 4 min after blue-light stimulation (1 s pulse duration) in the B nucleus. Note that B stimulation induced different responses in the S1 and mPFC cortices. The blue-light induced inhibition in mPFC lasted less than when the light was applied to the HDB. Examples of the SEP are shown on the right. **(G)** The increment of the SEP response in S1 cortex evoked by HDB stimulation was blocked by atropine sulfate (5 mg/kg) injected 10 min before blue-light stimulation (upper plot). The inhibition of SEP response in mPFC cortex evoked by HDB stimulation was blocked by mecamylamine (8 mg/kg; MEC) injected 10 min before blue light stimulation (lower plot). In **(E,F)** the mean of the two control values was considered as 100%. Inset in **(A)** indicates evoked potential area (green). ^∗^*p* < 0.05; ^∗∗^*p* < 0.001.

The above results suggest that HDB/VDB and B nuclei have a different cortical projection pattern that may differently modulate the cortical activity. To directly demonstrate this hypothesis, we decided to use optogenetic activation of BF neurons. We selectively drove the expression of high levels of channelrhodopsin-2, tagged with a fluorescent protein (ChR2-eYFP) in BF axons innervating the cortex (see Materials and Methods; **Figure 10C**). To allow protein expression and transport to the axon terminals, recordings were performed 3–4 weeks post-injection. At the end of recording sessions, animals were sacrificed and perfusion-fixed, and their brains processed for microscope analysis. As the viral vector used drives the simultaneous expression of ChR2 and the enhanced version of the yellow moiety of the Aequorea victoria fluorescent protein (eYFP), we ascertained the selective transfection of BF cells and their axons by immunolabeling the brain sections for eYFP (see **Figure 10C**). A dense population of neuronal cell somata and dendrites was observed at the injection site in the BF. In most of the experiments, this population was limited to the HDB/VDB or B nuclei, without invading into the adjacent BF areas; only these experiments were included in the optogenetic data analysis. Labeled axons were visible along their path in the white matter and terminal fields were studied in the mPFC. When the virus was injected in HDB, labeled axons were observed in layer 1 and numerous varicosities were mainly observed in layer 2/3 of the PL/IL (**Figure 10C**). In contrast, neither fibers nor terminals were observed in the PL/IL when the viral injection was located in the B nucleus (data not shown).

A blue-light pulse (1 s) able to stimulate a small volume of tissue (about 100–200 μm in radius; Aravanis et al., 2007) was delivered to the BF through a thin optic fiber (**Figures 10A,B**). A desynchronization of the local field potential recoded in S1 was observed (**Figure 10D**). Using this experimental setup, we tested the effect of optogenetic activation of HDB/VDB or B axons terminals on the somatosensory evoked potentials (SEPs) elicited by whisker stimulation in PL/IL and S1 cortices. The mean area of the earlier negative wave was calculated every 60 stimuli (1 min; **Figure 10E**, right traces). The control period consisted in 120 stimuli applied before blue light stimulation and the mean area was considered as 100%. Blue-light stimulation of HDB induced a SEP increase in S1 cortex. The SEP area increased rapidly, reaching a maximum 2 min after blue light stimulation (172 ± 25%, *P* = 0.018; *n* = 8) that was sustained until 4 min after stimulation (155 ± 27%; *P* = 0.042; *n* = 8). In contrast, SEP potentials were inhibited in the mPFC by blue-light stimulation when this was directed toward the HDB (**Figure 10E**). The mean area decreased slowly, reaching statistical significance 3 min after blue light stimulation (78 ± 8%, *P* = 0.004 and 83 ± 9%, *P* < 0.001, respectively; *n* = 8).

Blue-light stimulation of the B nucleus induced a lower SEP increase, reaching statistical significance at 3 and 4 min after blue light stimulation (121 ± 9%, *P* = 0.031 and 111 ± 5%, *P* = 0.048, respectively; *n* = 10; **Figure 10F**). Blue-light stimulation of the B nucleus also inhibited SEP in the mPFC (81 ± 5%, *P* < 0.001; *n* = 10). However, the stimulation effect vanished 2 min after optogenetic stimulation. In summary, data indicate that BF stimulation also has heterogeneous effects on the cortex according to the stimulation location, supporting the above anatomical data.

The plot of the percentage of SEP change induced by blue light stimulation respect to the control period is shown in **Figure 10G** 10 min after intraperitoneal injection of the muscarinic receptor antagonist, atropine, or mecamylamine, a nicotinic receptor antagonist. It is known that cholinergic receptors were blocked 10 min after intraperitoneal injection because the BF-evoked desynchronization of the S1 local field potential was blocked. Response facilitation induced in S1 cortex by HDB stimulation was blocked by atropine sulfate (5 mg/kg; i.p.; 84 ± 5%; *P* < 0.001; *n* = 6, 155 ± 27%; **Figure 10G**; upper plot). In contrast, inhibitory effects in mPFC evoked by HDB stimulation were not affected by atropine (data not shown) but were blocked by mecamylamine injection (8 mg/kg; i.p.; 110 ± 3%; *P* < 0.001; *n* = 6, 10 min after mecamylamine application respect to control values, 83 ± 9%; **Figure 10G**; lower plot).

## Discussion

**FIGURE 11:**
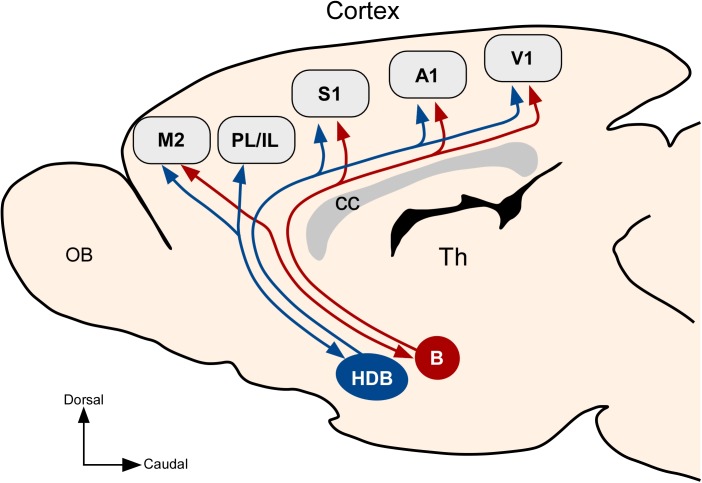
Summary diagram displaying anatomical connections of HDB and B nuclei. Results showed that HDB has preferential projections to sensory cortices and PL/IL cortices while the B nucleus projects to all sensory areas in general and does not project to PL/IL cortices but it projects to M2 cortex.

In this study, we tested the hypothesis of the existence of specific neuronal populations in the BF linking with specific sensory, motor and prefrontal cortices in rats. Our findings pointed out the presence of specific neuronal networks between the BF and the cortex that may play different roles in the control of cortical activity. Results suggest that the caudal part of the BF, including the B nucleus, shows less specificity in the projection to sensory cortical areas because this nucleus exhibits numerous double-labeled neurons. In contrast, VDH/HDB showed few double-labeled neurons when retrograde tracers were injected in primary sensory cortices, pointed out that they mainly project to a specific sensory cortical area, suggesting the existence of specific pathways that may modulate sensory processing in the cortex. In addition, we found reciprocity of the frontal cortical projections with the HDB/VDB and B nuclei that were not observed with sensory cortical areas (**Figure 11**). Using BF focal virus injections to express ChR2, we confirm anatomical findings because whisker responses were mainly modulated in S1 and mPFC by optogenetic stimulation of HDB neurons whereas changes in whisker responses after optogenetic stimulation of the B nucleus were less evident. Most of these effects were due to activation of the cholinergic neurons since they were blocked by intraperitoneally injected nicotinic (mecamylamine) or muscarinic (atropine) receptor antagonists. On the basis of these findings, control of cortical processing by cholinergic projections from the BF could be much more specific that it was traditionally thought.

Previous studies have described anatomical projections from the BF to different cortical areas (Bigl et al., 1982; Price and Stern, 1983; Houser et al., 1985; Zaborszky et al., 1986, 1997, 2005, 2015; Mesulam et al., 1992; Gritti et al., 1997, 2003; Butcher and Woolf, 2004; Nadasdy et al., 2010; Bloem et al., 2014; Kim et al., 2016). These studies have demonstrated a rostro-caudal distribution of neurons with different afferent and efferent inputs (also confirmed by present results). In addition, we provide novel information about the relationship between the location of BF neurons and their targets in the primary sensory and mPFC cortices. The HDB/VDB area is mainly related with primary sensory cortices with specific projections to S1, A1, or V1 according to the rostro-caudal neuronal distribution. In contrast, B nucleus projects to all sensory cortical areas without a clear specific pattern. Moreover, differences between HDB/VDB and B nuclei are also evident in their projections to mPFC. In fact, HDB/VDB, but no B nucleus, shows bidirectional projections to PL/IL cortices. In contrast, B nucleus projects to the M2 cortex while the projection from HDB/VDB to the M2 cortex is less important (**Figure 11**). Present data suggest that these BF areas are integrated in distinct BF-cortical networks that may play different roles in sensory processing, motor control, or cortical arousal.

The bidirectional connectivity between mPFC and BF has attracted great interest as a circuit involved in modulating decision making, cortical arousal, and learning and memory (Groenewegen and Uylings, 2000; Miller and Cohen, 2001; Dalley et al., 2004; Henny and Jones, 2008; Wise, 2008; Ramanathan et al., 2009; Martinowich et al., 2012; Paolone et al., 2012; Bloem et al., 2014; Zaborszky et al., 2015). The connections between the mPFC and other cortical areas that apparently contribute to information processing seem to be specific topographically organized (Hoover and Vertes, 2007; Bedwell et al., 2014, 2015; Zaborszky et al., 2015). In such processes, the cholinergic, GABAergic and glutamatergic BF neuronal groups or subgroups probably play diverse and complementary roles. Our results indicate that these projecting neurons are mainly concentrated in the rostral BF, including VDB and HDB. Their activation by optogenetic stimulation induced an important facilitation of whisker responses in S1, as has also been demonstrated in mice (Chaves-Coira et al., 2016). This response facilitation was due to activation of muscarinic receptors since it was blocked by the previous injection of atropine. Whisker responses have been also recorded in the mPFC (Martin-Cortecero and Nuñez, 2016). However, the same BF projection inhibited whisker responses in mPFC though activation of nicotinic receptors since the inhibition was abolished by mecamylamine injection. We cannot discard that non-cholinergic neurons in the BF may also contribute to modulate sensory responses in the cortex, as has been indicated previously (Duque et al., 2000; Zaborszky et al., 2015).

This BF-mPFC anatomical pathway seems to be specific since projections to cortical areas mainly arise from specific BF neuronal groups, as supported by the observation that most of the HDB neurons were single-labeled after cortical injection of retrograde tracers. Accordingly, retrograde tracing in mice has shown that very few V1-projecting cholinergic BF neurons project to other cortical areas (Pinto et al., 2013). Thus, it is reasonable to think that the pathway linking HDB-primary sensory cortices-mPFC (through an indirect pathway) and back to BF may be relevant in the specific sensory processing that is required for attention or learning processes.

In contrast, using the same experimental protocol of retrograde tracer injection, the B nucleus showed a large proportion of double-labeled cortical neurons, suggesting that the B nucleus is non-specifically connected to sensory areas and is not directly related with mPFC. However, HDB and B nuclei are also connected to the M2 cortex. The rostral part of M2 has been included as a different subregion of PFC that may contribute to sensory-motor integration, linking sensory information to motor actions (Barthas and Kwan, 2017). Accordingly, the anatomical specificity described here has also been observed in the neuronal activation of prefrontal cortex-projecting vs. motor cortex-projecting BF neurons during task performance (Parikh et al., 2007). Thus, B nucleus neurons may integrate information necessary for sensory-motor modulation from the striatum (Hu et al., 2016).

The BF is recognized as an important site for sleep-wake regulation and the control of arousal level (e.g., Koyama, 2012; Kalinchuk et al., 2015); actually, BF receives wide-spread input from hypothalamic nuclei, including the preoptic areas and lateral hypothalamic area, both of which are implicated in sleep and arousal regulation (Szymusiak et al., 2007; Agostinelli et al., 2016; Hu et al., 2016). Considering the strong reciprocal connections between BF and hypocretinergic neurons our results could suggest that B nucleus could be a good candidate to promote arousal.

## Conclusion

In conclusion, complex behaviors require the integration of different synaptic inputs in the brain. These functions cannot be performed using the traditional description of the forebrain cholinergic system as a diffusely organized neuromodulator system with widespread influence on information processing across large portions of the cortex. Recent evidence supports an alternative hypothesis that proposes that the cognitive functions of cholinergic projections are determined in part by BF-cortical circuitry controlling cholinergic synaptic neurotransmission release in a more specific manner than previously assumed (Hasselmo and Sarter, 2011; Zaborszky et al., 2015). Our results agree with this alternative hypothesis and further suggest that the cortex and BF are integrated in specific neuronal networks with different roles in sensory processing, motor planning or arousal control.

## Author Contributions

MR-A and AN conceived and supervised all aspects of the study. IC-C and JM-C collected all data. IC-C analyzed anatomical aspects of the data. JM-C analyzed electrophysiological aspects of the data.

## Conflict of Interest Statement

The authors declare that the research was conducted in the absence of any commercial or financial relationships that could be construed as a potential conflict of interest.
